# Chrononutritional Effects of Cherry Consumption on Hepatic Lipid Profile

**DOI:** 10.3390/nu18020345

**Published:** 2026-01-21

**Authors:** Maria Josefina Ruiz de Azua, Francesca Manocchio, Álvaro Cruz-Carrión, Anna Arola-Arnal, Carolina Gerstner, Claudio Bernal, Manuel Suárez

**Affiliations:** 1 Universitat Rovira i Virgili, Departament de Bioquímica i Biotecnologia, Nutrigenomics Research Group, 43007 Tarragona, Spain; josefina.ruizdeazua@eurecat.org (M.J.R.d.A.); francesca.manocchio@upf.edu (F.M.); alvarocruzcarrion@gmail.com (Á.C.-C.);; 2 Nutrigenomics Research Group, Institut d’Investigació Sanitària Pere Virgili, C/Marcel·lí Domingo 1, 43007 Tarragona, Spain; 3 Center of Environmental Food and Toxicological Technology (TecnATox), C/Marcel·lí Domingo 1, 43007 Tarragona, Spain; 4 Cátedra Bromatología y Nutrición, Facultad de Bioquímica y Ciencias Biológicas, Universidad Nacional del Litoral, Santa Fe 3000, Argentinacbernal@fbcb.unl.edu.ar (C.B.)

**Keywords:** fatty acids, photoperiod, (poly)phenols, seasonality

## Abstract

**Background**: Consumption of fruits of different origins with specific (poly)phenolic profiles can modulate the gene expression of enzymes and the levels of metabolites in a photoperiod-dependent manner. However, there is little information on how this affects the profile of hepatic and muscular fatty acids (FAs) and how it interferes with metabolic pathways. This study aimed to determine whether consuming local or non-local cherries alters liver and muscle FA profiles under different photoperiods, and to identify the associated changes in metabolic gene expression and serum metabolites. **Methods**: Seventy-two Fischer 344 rats, fed a standard diet and either vehicle (VH), Local Cherry (LC), or non-Local Cherry (nLC), were exposed to different hours of light to simulate photoperiods (winter, spring/autumn, or summer) for 7 weeks. The FA profiles of the liver and muscle were determined using GC-FID, and the gene expression of key enzymes involved in FA metabolism was evaluated. Moreover, the composition of hydrophilic and lipophilic metabolites in the serum and liver was analyzed using nuclear magnetic resonance (NMR), and pathway analysis was performed. **Results**: Consumption of cherries in season (18 h of light) decreased saturated FAs levels in the liver, mainly palmitate, compared to their respective VH; interestingly, this effect was not observed in other photoperiods. Furthermore, muscle polyunsaturated FA (PUFAs) decreased, possibly due to increased oxidation. **Conclusions**: Seasonal cherry consumption improves the hepatic lipid profile and increases muscular oxidation. Future studies are needed to better define these effects and uncover the differences in lipid metabolism in response to cherry consumption.

## 1. Introduction

Fatty acids (FAs) have different physicochemical and biological properties depending on the length of the carbon chain and the number and configuration of double bonds [1,2]. They are divided into saturated fatty acids (SFAs), monounsaturated fatty acids (MUFAs), and polyunsaturated fatty acids (PUFAs), including *n*-3 and *n*-6 PUFAs [3]. Furthermore, FAs can be part of different complex lipids, such as glycerolipids, glycerophospholipids, and sphingolipids. The importance of FAs lies not only in their role as energy substrates but also in their key relevance as modulators of metabolic and inflammatory processes. For instance, they act as lipid mediators, cell signaling molecules, and modulators of the expression of some genes [2]. MUFAs and PUFAs are thought to benefit serum lipid profiles by limiting hepatic steatosis, subsequently increasing lipid metabolism and decreasing lipogenesis. The liver is a key tissue involved in FA metabolism, where FA oxidation, synthesis, elongation, and desaturation occur [4]. In this regard, the enzymes Δ6 and Δ5 desaturases (FADS2 and FADS1, respectively) play key roles in PUFA synthesis. Moreover, there is crosstalk between the liver and skeletal muscle, as muscle cells are also involved in lipid oxidation [5]. Several factors influence hepatic lipid metabolism. Specifically, FADS1 and FADS2 have been shown to be regulated by nutritional, hormonal, and genetic factors. For instance, diets with high protein content or deficiencies in essential FAs stimulate FADS2 activity, whereas a high intake of glucose, fructose, or low protein suppresses it [6]. Interestingly, some authors have observed that (poly)phenols can influence the activity of these enzymes [7]. In this regard, the activity of FADS2 is inhibited or stimulated depending on the nature of the (poly)phenols. For example, quercetin induces the expression of *Fads1*, whereas apigenin and fisetin inhibit it [8]. A stimulatory effect of resveratrol on *Fads1* and *Fads2* mRNA concentrations has also been observed [9].

(Poly)phenol consumption is associated with anti-inflammatory, antioxidant, anticarcinogenic, antidiabetic and lipid-lowering properties, among others [10,11]. These effects are due to the modulation of key metabolic pathways by different mechanisms. Therefore, phenol-rich diets, with a high consumption of products with high (poly)phenolic content, such as the Mediterranean diet, have shown beneficial effects on consumers [12]. Among these phenol-rich foods, cherries are characterized by their high flavonoid content, including anthocyanins and hydroxycinnamic acids. Its intake has been associated with a decreased risk of chronic diseases [13]. For example, preclinical studies have demonstrated that sweet cherry consumption counteracts the detrimental effects of metabolic disorders induced by a diet high in sugars [14].

According to the xenohormesis theory, the consumption of (poly)phenols is a way for heterotrophs to adapt to their environment and increase their survival. As the content of phytochemicals is dependent on agronomic factors, including location, as well as postharvest treatments, the consumption of plants from different origins may provide specific signals that would impact human health [15,16]. Additionally, biological rhythms, including circadian and circannual rhythms, influence the effects of food consumption [17,18]. This interplay influences systemic and brain health; indeed, the timing of (poly)phenol intake may critically modulate their metabolism, bioactivity, and neuroprotective efficacy [19,20]. In our previous studies we observed that cherry consumption differentially influences the serum concentrations of triacylglycerides (TAGs) and non-esterified FAs (NEFAs), as well as the oxidative state and gene expression of certain liver enzymes, depending on the origin and photoperiod (hours of light per day) [21,22]. A recent study also showed that not only the whole fruit but also fruit extracts, including those from cherries, modulate TAGs levels in a photoperiod-dependent manner [23]. Although previous studies have shown that cherry consumption and photoperiod modulate metabolic pathways, it remains unclear how these factors interact to shape the hepatic and muscular FA profiles. This gap limits our understanding of the chrononutritional effects of cherry consumption, which the present study aimed to address. Moreover, there is limited information on how these factors influence the FAs composition of metabolically active tissues such as the liver and muscles. Therefore, in the present study we also aimed to characterize the hepatic and muscular FA profiles of Fischer 344 rats exposed to different hours of daily light supplemented with cherries of different origins. We also provide evidence of changes in key hepatic and serum hydro- and lipophilic metabolites.

## 2. Materials and Methods

### 2.1. Fruits Preparation

*Brooks* sweet cherries grown and harvested in different geographic regions (Tarragona, Spain (LC, Local-Cherry) and Cachapoal, Chile, (nLC, non-Local Cherry)) were used. LC were kindly donated by farmers of Camp de Tarragona in June, while nLCs were purchased from a local market in December. After discarding the pips, the cherries were frozen in liquid nitrogen, ground, and lyophilized at −55 °C (Telstar LyoQuest lyophilizer, Thermo Fisher Scientific, Barcelona, Spain). The nutritional compositions of both fruits have been previously characterized [22]. Briefly, LC had a significantly higher content of protein, total lipids, dietary fiber, total (poly)phenols, anthocyanins, and flavanols, while nLC had a higher content of total carbohydrates, sugars, and flavonols [22].

### 2.2. Animals and Experimental Design

Seventy-two 8-week-old male Fischer 344 (F344) rats were housed in pairs at 22 °C. To simulate the days of the different annual seasons, the animals were divided into three photoperiods with different hours of light per day (light density 700 lx): short-day (*n* = 24; L6, 6 h of light), standard (*n* = 24; L12, 12 h of light), or long-day (*n* = 24; L18, 18 h). After 4 weeks of adaptation, within each photoperiod the animals were randomly divided according to the treatment received: vehicle (VH, *n* = 8), LC (*n* = 8), or nLC (*n* = 8). This strain was chosen based on previous studies showing that it is sensitive to photoperiod, as it exhibits changes in body weight, energy balance and metabolic adaptations [24,25]. The animals were fed a standard diet (STD) (A04 SAFE; Panlab, Barcelona, Spain). One hour after the light was turned on, the treated animals were administered VH or a dose of 100 mg/kg body weight of freeze-dried fruit (diluted in water). The cherry dose was selected based on the results of a previous study [26]. The VH consisted of a sugar solution (glucose/fructose 1:1, 21.2 mg per kg of body weight) to equal sugar content administered to the animals treated with cherries. The amount of kcal provided by the VH represents only 0.11% of daily energy; therefore the effect of the sugar load is not considered to have a relevant impact [22]. To guarantee complete intake of the dose, the treatments were administered by voluntary licking through a syringe. After 7 weeks of treatment, the animals were sacrificed by decapitation, and the last dose one hour before sacrifice. Blood was collected in non-heparinized tubes, incubated for 1 h at room temperature, and immediately centrifuged at 1200× *g* for 15 min to collect the serum. Liver, muscle, and serum samples were stored at −80 °C until further analysis.

### 2.3. Analytical Determinations

The gene expression of enzymes related to lipid metabolism in the gastrocnemius muscle and liver was analyzed using standardized protocols, including RNA extraction, subsequent cDNA synthesis, and amplification by real-time polymerase chain reaction (RT-qPCR).

Total RNA was extracted from the liver and gastrocnemius muscle using TriPure reagent (Roche Diagnostics, Sant Cugat del Vallès, Barcelona, Spain) according to the manufacturer’s protocol. cDNA was synthesized by Reverse Transcription using High-Capacity cDNA Reverse Transcription (Thermo Fisher Scientific, Illkirch-Graffenstaden, France). Specific cDNA amplification was performed using real-time polymerase chain reaction (RT-qPCR) using iTaq Universal SYBR Green Supermix (Bio-Rad, Barcelona, Spain). Eight samples were used per group (*n* = 8). Different primers related to hepatic metabolism of FAs and muscle lipid metabolism, obtained from Biomers.net (Ulm, Germany) were used (Supplementary Table S1): *Acc2*, acetyl-CoA carboxylase 2; *AdipoR2*, Adiponectin receptor 2; *CS*, Citrate Synthase; *Elovl2*, elongation of very-long-chain fatty acid enzyme 2; *Fads1*, Δ-5 desaturase; *Fads2*, Δ-6 desaturase; *FAT*/*cd36*, fatty acid translocase, homologue of CD36; *Scd1*, Δ-9 desaturase. The relative expression of each mRNA was calculated as a percentage of the L18 group using the Pfaffl method. In addition, the FA profiles of these tissues and the corresponding enzymatic fluxes were analyzed by gas chromatography coupled to a FID detector (GC-FID), following previously described protocols [27,28,29]. The methodology of these procedures is comprehensively described in the Supplementary Material. Finally, the hydrophilic and lipophilic metabolites in the serum and liver were analyzed using nuclear magnetic resonance (NMR). Extraction was performed following the protocol described by Palacios-Jordan et al. (2020), with some modifications (Supplementary Material) [30].

### 2.4. Statistical and Multivariate Analysis

Homogeneity was evaluated using Levene’s test, as well as the normality of the data. One- and two-way ANOVA and post hoc DMS tests were performed to determine the effect of the main factors and differences between the experimental groups. Student’s *t*-test was used to identify differences between two groups. Statistical Product and Service Solutions (SPSS) software, version 22 (SPSS Inc., Chicago, IL, USA) was used to perform the analyses. Statistical significance was set at *p* < 0.05 and *p*-values between 0.05 and 0.1 were considered as trends. Partial least squares-discriminant analysis (PLS-DA) was conducted as a supervised method to identify the hydrophilic and lipophilic metabolites with higher discriminative power in serum and liver. Variable importance in projection (VIP) scores (>1) was used to evaluate the significance of these metabolites. R2Y (the interpretability of the model) and Q2 (the predictability of the model) were used to verify the suitability and reliability of the PLS-DA model. A permutation test per 1000 was applied to further check the validity of the model. Additionally, pathway analysis was performed using MetaboAnalyst (version 5.0) to determine the overall metabolic influence of exposure to different photoperiods. *p*-values and false discovery rates (FDRs) were used to verify the affected pathways.

## 3. Results

### 3.1. Seasonal Consumption of Cherries Decreased the Total Hepatic SFA Content

**Figure 1:**
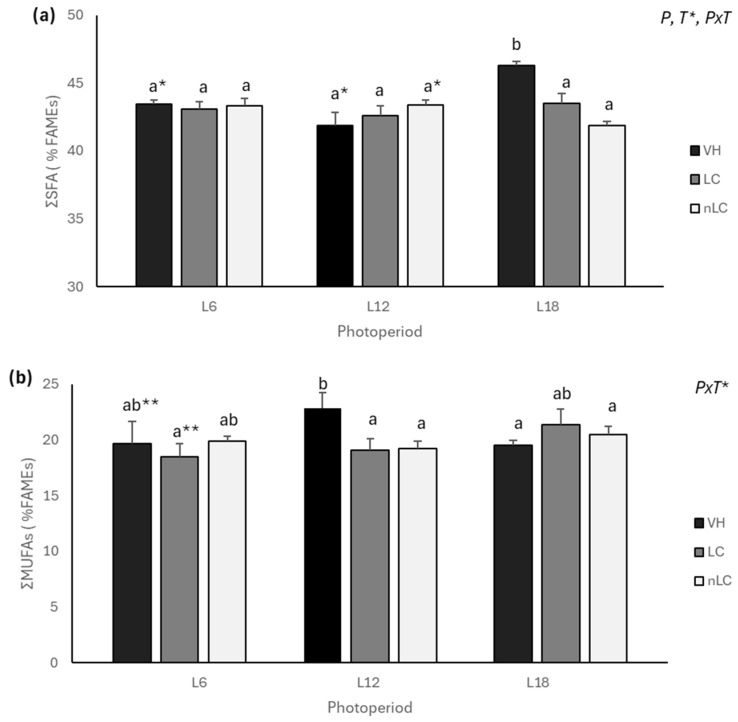
Proportion of saturated fatty acids (**a**) and monounsaturated fatty acids (**b**) expressed as % FAMEs in the liver of animals exposed to different photoperiods (L6, L12, and L18 correspond to 6, 12, and 18 h of light per day, respectively) and supplemented with treatment: Local Cherry (LC), non-Local Cherry (nLC), or vehicle (VH). ΣSFAs: saturated fatty acids; ΣMUFAs: monounsaturated fatty acids. Values expressed as mean ± SEM (*n* = 4). P, photoperiod; T, treatment; PxT, interaction of photoperiod and treatment. (Two-way ANOVA (2xA), *p* < 0.05); different letters (a, b) indicate significant statistical differences *p* < 0.05; * indicates trend 0.05 < *p* < 0.1 (post hoc DMS, one-way ANOVA). ** Indicates trend (0.05 < *p* < 0.1) in the same photoperiod (Student’s *t*-test).

Table 1 shows the % Fatty Acid Methyl Esters (FAMEs) of the main hepatic FAs, while Supplementary Table S2 shows all the FAs analyzed. The results showed that both photoperiod and its interaction with the treatment significantly affected the proportion of hepatic SFA (Figure 1a). Remarkably, cherry consumption in season (L18), regardless of origin, decreased SFA levels compared to the VH. This difference is mainly due to a 6.03% and 9.51% lower palmitic acid (PA) content in LC and nLC, respectively. The L18-nLC group also showed a trend towards a lower level of stearic acid (SA) than the VH group (*p* = 0.062). Interestingly, L12 animals showed a higher level of SA than their respective VH (Supplementary Material Table S2). At the muscular level, the treatment significantly affected the proportion of SFAs, whereas the interaction of photoperiod with the treatment showed a trend (*PxT*, *p* = 0.086). Table 2 shows the % FAMEs of the main muscle FAs, while Supplementary Table S3 shows all FAs analyzed. Specifically, the L12-nLC and L6-LC groups presented a higher proportion of SFAs than their respective VH. In particular, the L12-nLC presented more SA and less 14:0 FA, whereas L6-LC animals tended to have a greater amount of PA than their VH (*p* = 0.058). Interestingly, L18-LC animals, despite not having significant changes in SFAs between treatments, presented with a lower amount of SA.

### 3.2. Cherry Consumption Did Not Affect the Content Liver MUFAs Content

The results showed a trend towards an interaction between photoperiod and treatment, affecting the ratio of hepatic MUFAs (*PxT*, *p* = 0.078). In this sense, cherry consumption, both LC and nLC, decreased MUFAs in animals exposed to L12 (Figure 1b), mainly due to a decrease in oleic acid (OA) (Supplementary Material Table S2). Likewise, the consumption of LC in L6 also tended to decrease MUFAs with respect to VH (*p* = 0.085), with palmitoleic acid being the FA significantly affected. Although the treatments did not alter the MUFAs in the animals exposed to L18, in both LC and nLC treated groups, the proportion of c11-18:1 increased significantly by 13.38% and 17.06%, respectively. It was also observed that the L18-nLC group increased the level of c11-20:1 with respect to its VH (Supplementary Table S2).

**Figure 2:**
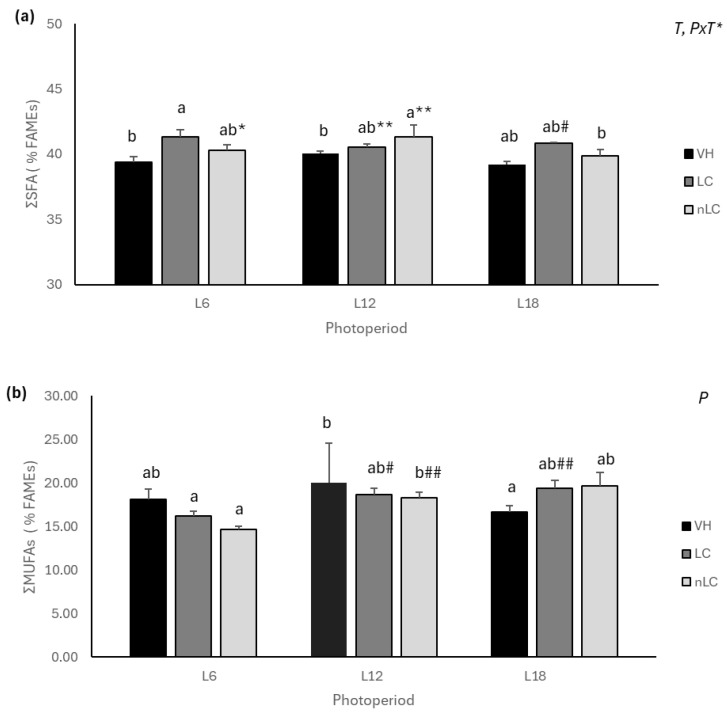
Proportion of saturated fatty acids (**a**) and monounsaturated fatty acids (**b**) expressed as % FAMEs, in muscle of animals exposed to different photoperiods (L6, L12, and L18 correspond to 6, 12, and 18 h of light per day, respectively) and supplemented with treatment: Local Cherry (LC), non-Local Cherry (nLC), or vehicle (VH). ΣSFA: saturated fatty acids; ΣMUFAs: monounsaturated fatty acids. Values expressed as mean ± SEM (*n* = 4). P, photoperiod; T, treatment; PxT, interaction of photoperiod and treatment. (Two-way ANOVA (2xA), *p* < 0.05); different letters (a, b) indicate significant statistical differences *p* < 0.05; * indicates trend 0.05 < *p* < 0.1 (post hoc DMS, one-way ANOVA). ** Indicates trend (0.05 < *p* < 0.1) in the same photoperiod. # Indicates significant statistical differences *p* < 0.05 and ## indicates trend 0.05 < *p* < 0.1 (Student’s *t*-test).

Figure 2b shows that photoperiod significantly affected the proportion of muscle MUFAs. Specifically, animals that consumed LC in season tended to increase them with respect to their VH (*p* = 0.064: Student’s *t*-test), while those exposed to L12 decreased it. In particular, the L18-LC group showed an increase in c11-18:1 and a tendency toward higher palmitoleic acid levels (*p* = 0.087, Student’s *t*-test), whereas those exposed to L12 showed the opposite effect (Supplementary Material Table S2).

### 3.3. Muscle PUFAs Were Decreased by LC Consumption During Season

The treatment significantly affected the levels of total, *n*-6, and *n*-3 hepatic PUFAs, the latter also being affected by the interaction of photoperiod and treatment (*PxT*, *p* = 0.087) (Table 1). Specifically, L18-nLC animals showed an increase in total PUFAs and *n*-6 PUFAs. Moreover, these animals showed a higher level of c11, c14-20:2, linoleic acid (LA), α-linolenic acid (ALA) and a lower proportion of eicosapentaenoic acid (EPA). However, the animals in the L18-LC group did not show any differences compared to the VH group.

The groups that received cherries in L12, regardless of their origin, showed higher levels of hepatic total PUFAs and *n*-3 PUFAs than the control group. The treated animals showed a higher proportion of docosahexaenoic acid (DHA) and arachidonic acid (AA) than the VH group. In addition, the animals that received nLC had 36.14% less ALA whereas they had 35.95% more gamma-linolenic acid (GLA). This effect was not observed in the L12-LC animals. It should be noted that the animals exposed to L6 did not show significant changes in the sums of the different PUFAs, or in hepatic FA.

As shown in Table 2, at the muscular level, total PUFAs decreased in animals treated with cherry at L18. Interestingly, the L18-LC group also had lower levels of PUFAs *n*-3 and *n*-6. Specifically, these animals tended to have lower DHA levels than their VH (*p* = 0.076). The L18-nLC group also tended to have a lower proportion of EPA (*p* = 0.069, Student’s *t*-test). In contrast, the L12-LC group not only had a higher level of DHA than the VH group but also showed a trend towards more AA (*p* = 0.089) and c7,c10,c13,c16-22:4 (*p* = 0.081) levels. The consumption of cherries in L12 not only increased the total PUFA content but also the amount of *n*-6. Furthermore, L12-nLC presented a lower level of LA than that of VH. In L6 animals, no significant changes were observed in the PUFA levels.

### 3.4. Seasonal LC Consumption Increases the Unsaturation Flux Through Scd1

Table 1 also shows the flux of the enzymatic metabolism estimating the values of some key hepatic enzymes related to the conversion rate of LC-PUFAs, expressed as product/substrate ratios. In season, the L18-LC animals tended to have higher values of the *Scd1* enzyme flux than the corresponding VH animals, which can be estimated from the ratio between palmitoleic acid and PA (*p* = 0.079, Student’s *t*-test). In contrast, no such effect was observed in animals that ingested nLC. Interestingly, out of season, the groups that received LC and were exposed to L6 and L12 showed an opposite flux, with their respective VH showing a higher flux. Moreover, *Scd1* produces oleic acid through the unsaturation of stearic acid. Therefore, we estimated the enzyme flux based on the OA/EA ratio. The interaction between photoperiod and treatment significantly affected the flux through this enzyme. Although no significant changes were observed in the animals exposed to L18 or L6, those that consumed cherries in L12 showed statistically lower values of this parameter with respect to the VH.

The interaction between photoperiod and treatment tended to affect the DHA/ALA and AA/LA ratios, which are indicators of the biosynthesis of PUFAs *n*-3 and *n*-6, respectively *(PxT*, *p* = 0.055; *p* = 0.087, two-way ANOVA, respectively). In this sense, the L12-nLC animals showed statistically higher values than their respective VH values for both parameters. The evaluated treatments also tended to significantly affect the flux through the enzyme elongase, estimated from the c11-18:1/palmitoleic acid ratio, an indicator of de novo FA biosynthesis (*T*, *p* = 0.055, two-way ANOVA). Specifically, the L18-nLC animals showed statistically higher values than their respective VH values. In addition, the consumption of LC had the same effect on the animals (*p* = 0.062).

### 3.5. Consumption of nLC in the Season Mainly Affected the Metabolism of LA, Whereas the Intake of LC Did Not Produce Any Changes

**Figure 3:**
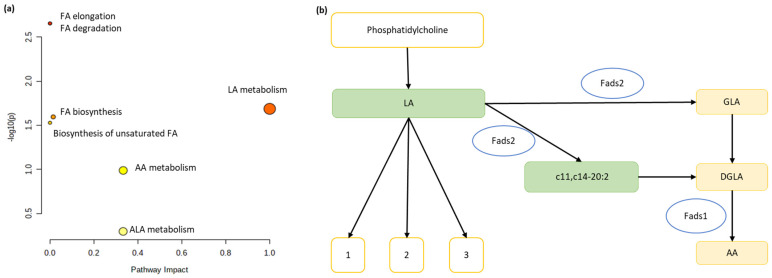
**Pathway analysis based on data from twenty-seven hepatic fatty acids (FAs) of the L18-nLC vs. L18-VH groups**. Overview of pathway analysis (**a**) and pathway of linoleic acid metabolism (**b**) from Kyoto Encyclopedia of Genes and Genomes (KEGG) database. Green boxes indicate increase in FA in L18-nLC animals; white boxes indicate no changes in lipid classes. Image elements: 1, (9R,10S)-(12Z)-9,10-Epoxyoctadecenoic acid; 2, (12R,13S)-(9Z)-12,13-Epoxyoctadecenoic acid; 3, (9Z,11E)-(13S)-13-Hydroperoxyoctadeca-9,11-dienoic acid; AA, arachidonic acid: c5,c8,c11,c14-20:4 *n*-6; LA, Linoleic acid: c9,c12-18:2; ALA, α-linolenic acid: c9,c12,c15-18:3 *n*-3; DGLA, Dihomo-γ-linolenic acid: c8,c11,c14-20:3; GLA, gamma-linolenic acid: c6,c-9,c12-18:3 *n*-6; Fads1; Δ-5 desaturase; Fads2, Δ-6 desaturase.

In light of the significant changes in the FA profiles of the liver and muscle of the treated animals, we performed a pathway analysis to clarify the affected pathways during the usual season of cherry consumption, L18. Supplementary Table S4 shows the main metabolic pathways that exhibited significant changes when comparing animals treated with nLC and VH. Although several pathways seemed to be affected, only LA metabolism in nLC animals had a significant FDR and an impact of 1, meaning that the number of metabolites affected among the groups studied was significant in influencing the metabolic pathway. In Figure 3a and Figure 3b, an overview of the pathway analysis and detailed pathway of LA metabolism are shown, respectively. However, no significant alterations were observed in any metabolic pathway when performing pathway analysis between the L18-LC and L18-VH groups (Supplementary Table S4 and Supplementary Figure S1).

### 3.6. Consumption of LC in Season Increased the Gene Expression of Hepatic Scd1 and Muscle AdipoR2 Levels

**Figure 4:**
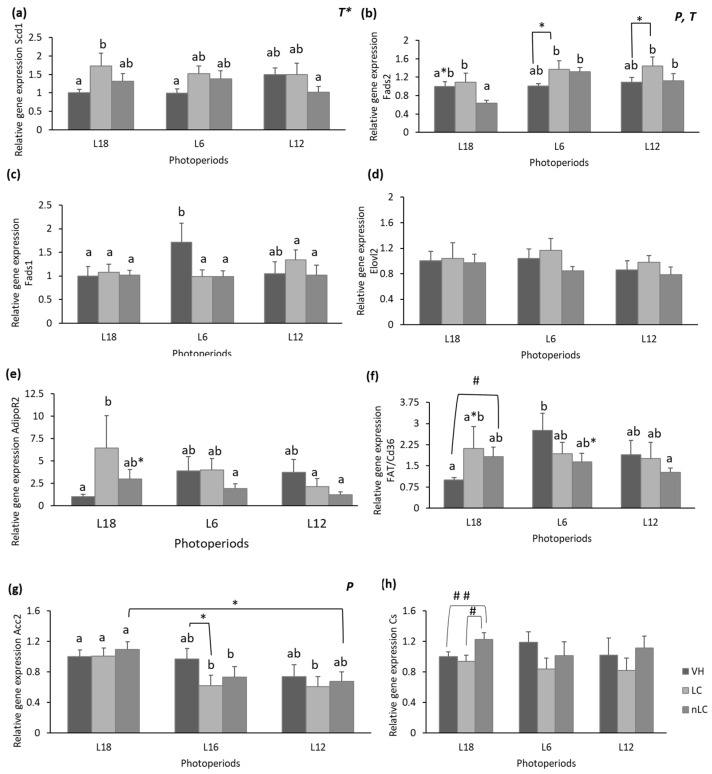
Gene expression of Δ-9 desaturase (*Scd1*) (**a**), Δ-6 desaturase (*Fads2*) (**b**), and Δ-5 desaturase (*Fads1*) (**c**) and elongation of very-long-chain fatty acid enzyme 2 (*Elovl2*) (**d**) in liver, and gene expression of Adiponectin receptor 2 (*AdipoR2*) (**e**), fatty acid translocase, homologue of CD36 (*FAT*/*cd36*) (**f**), Acetyl-CoA carboxylase 2 (*Acc2*) (**g**), and Citrate Synthase (*Cs*) (**h**) in gastrocnemius muscle of male Fischer 344 rats. The animals were treated for 7 weeks with vehicle (VH), Local (LC), or non-Local Cherry (nLC), and exposed to different photoperiods (short; L6, standard; L12 or long; L18). Values expressed as mean ± SEM (*n* = 8). The values were normalized by the L18-VH group. P, photoperiod effect; T, treatment effect (two-way ANOVA, *p* < 0.05). Different letters above the bars indicate significant differences (*p* < 0.05), while * indicates trend (0.05 < *p* < 0.1) (post hoc DMS, one-way ANOVA). # Indicate significant differences (*p* < 0.05, Student’s *t*-test) and ## indicates trend (0.05 < *p* < 0.1, Student’s *t*-test).

Due to the changes observed in the lipid profile, enzyme flux, and FAs pathways, we performed a gene expression analysis of key hepatic enzymes. As shown in Figure 4a, the hepatic expression of *Scd1* tends to be affected by the treatment (*T*, *p* = 0.057, two-way ANOVA), since the consumption of LC in season increased it significantly with respect to their VH. However, this effect was not observed when the animals ingested it at L12 or L6, or when they consumed nLC at L18. Therefore, these results point to a differential effect of the consumption of locally produced cherries in season over their consumption out of season compared to their respective VH or even the consumption of cherries from different geographical origins.

On the other hand, hepatic *Fads2* mRNA concentration was affected by both photoperiod and treatment, since L18-nLC animals showed lower expression than L18-LC and lower than VH (*p* = 0.072) (Figure 4b). However, this effect was not been observed out of season. In contrast, at L6 and L12, LC consumption tended to increase the gene expression of this desaturase compared to their respective VH (*p* = 0.067 and *p* = 0.086, respectively).

Figure 4c shows that the gene expression of *Fads1* decreased with cherry consumption, regardless of its origin, in L6. However, no statistically significant effects of treatment, photoperiod, or the intersection of both were observed using two-way ANOVA. The expression of *Elovl2* was not modified in any of the experimental groups (Figure 4d).

Due to the fact that the gastrocnemius muscle is one of the main catabolic tissues of FAs, we analyzed the gene expression of transporters and key genes involved in the oxidation processes. The results showed that the gene expression of gastrocnemius muscle enzymes and transporters was affected differently. Specifically, the mRNA concentration of *AdipoR2* was significantly increased in animals that consumed LC compared to those that ingested VH (Figure 4e). This differential effect of the treatment was not observed in the other photoperiods; even in L12, the behavior of the animals was completely the opposite, although it did not reach statistical significance. The L18-nLC animals showed increased gene expression of the muscle FA transporter, *FAT*/*cd36*, compared to their respective VH. In contrast, VH exposed to L6 showed a trend of higher mRNA expression than those that consume nLC (*p* = 0.078) (Figure 4f).

Additionally, photoperiod significantly affected the muscle gene expression of *Acc2*. As observed in Figure 4g, the animals exposed to L18 and consumed cherry showed a higher concentration of mRNA of the enzyme than those exposed to L6 or L12. It should be noted that the L6-LC group tended to present a lower gene expression than its respective VH (*p* = 0.065).

In contrast, the mRNA concentration of Citrate Synthase (*Cs*) increased significantly in the animals that received nLC compared to those that received LC (Figure 4h). In addition, they tended to have a higher expression in relation to their respective VH (*p* = 0.067, Student’s *t*-test).

### 3.7. Multivariate Analysis Showed a Differential Effect on Serum Metabolite Homeostasis Between Treatments at Different Photoperiods

**Figure 5:**
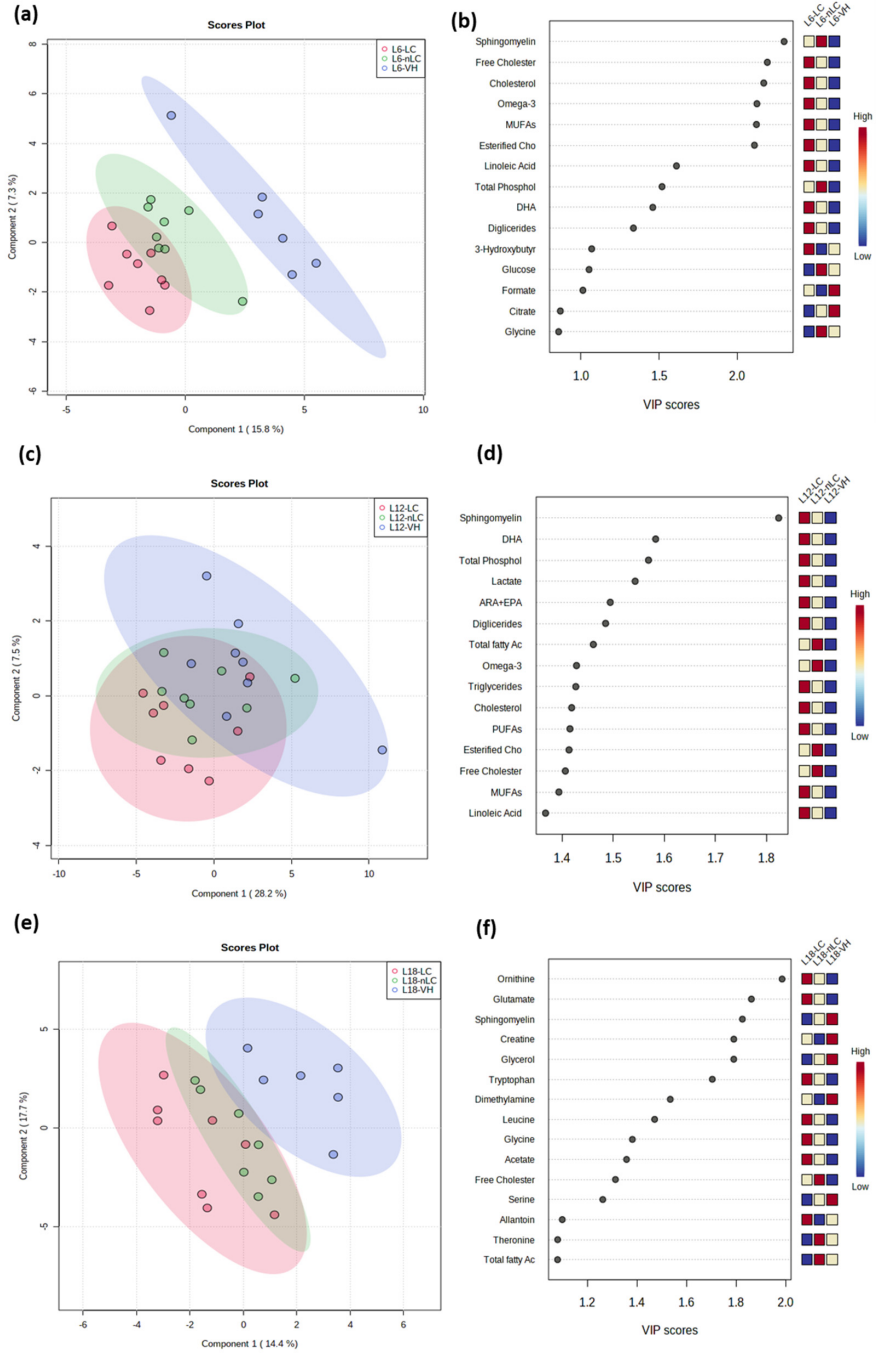
Statistical analyses of forty-nine metabolites (hydrophilic and lipophilic) in serum rats Fischer 344 fed with vehicle (VH), Local Cherry (LC), or non-Local Cherry (nLC), and exposed to different photoperiod with 6 (L6), 12 (L12), or 18 (L18) hours of light by Metaboanalyst 5.0 software. The figure images (**a**,**c**,**e**) show the PLS-DA score plot and the figure images (**b**,**d**,**f**) show the VIP scores for animals exposed to L6, L12, and L18, respectively. The score received, from low to high, determines the importance of the variables. The boxes on the right show the relative concentration of each of the metabolites. Red indicates high levels, and blue indicates low levels.

**Figure 6:**
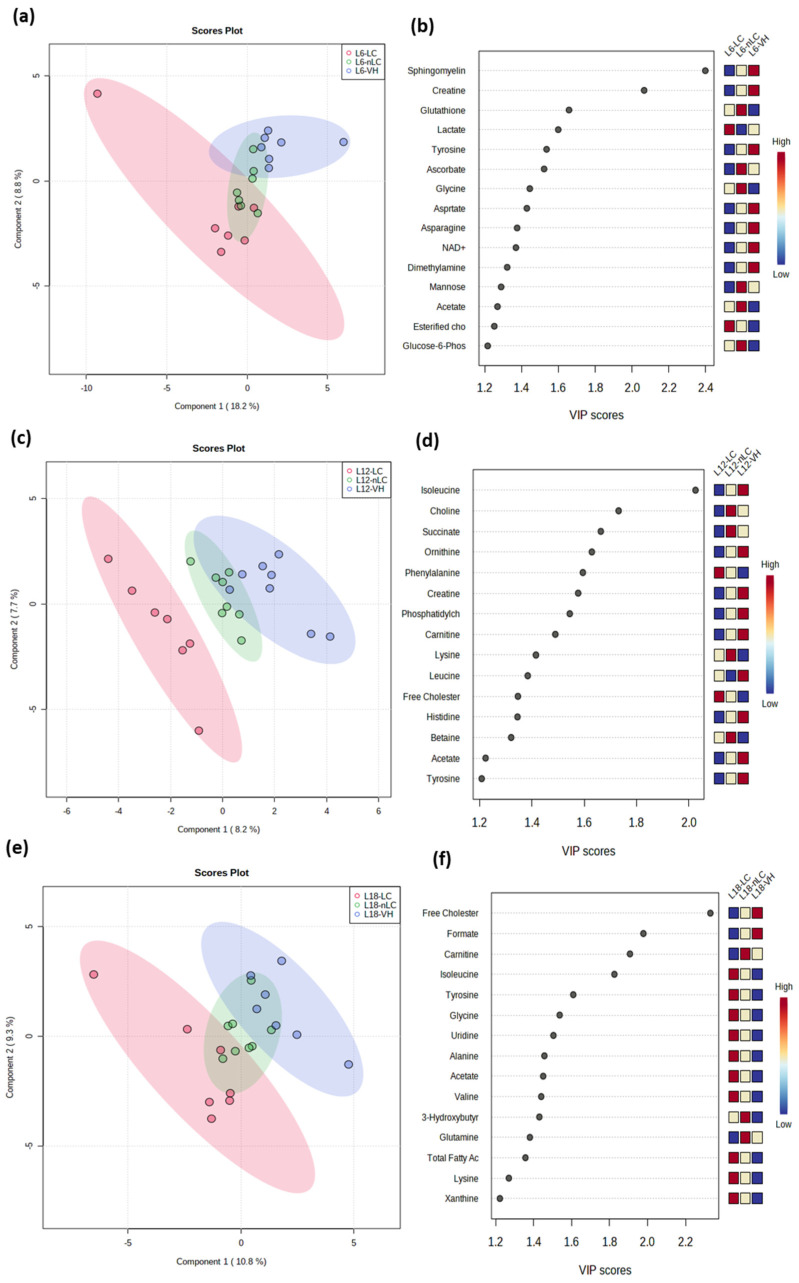
Statistical analyses of fifty-four metabolites (hydrophilic and lipophilic) in liver rats Fischer 344 fed with vehicle (VH), Local Cherry (LC), or non-Local Cherry (nLC), and exposed to different photoperiods with 6 (L6), 12 (L12), or 18 (L18) hours of light by Metaboanalyst 5.0 software. The figures (**a**,**c**,**e**) show the PLS-DA score plot and the figure images (**b**,**d**,**f**) show the VIP scores for animals exposed to L6, L12, and L18, respectively. The score they receive from low to high determines the importance of variables. The colored boxes on the right show the relative concentration of each of the metabolites. Red indicates high levels, and blue indicates low levels.

Due to the observed changes in hepatic and muscle FA profiles and the gene expression of enzymes and metabolic pathways, we decided to study the hydrophilic and lipophilic metabolites in both serum and liver to unravel the mechanisms involved in the observed effects. From the list of metabolites evaluated by NMR, after alignment and normalization of the spectra, 49 and 54 serum and liver metabolites were found in the samples, respectively, which are detailed in Supplementary Table S5. To determine how cherry type influenced the homeostatic balance of metabolites depending on the photoperiod, we first analyzed the hydrophilic and lipophilic metabolites in the serum (Figure 5) and in liver (Figure 6) using PLS-DA. Notably, when analyzing the serum metabolites, a clear grouping of the animals that consumed cherry, both LC and nLC, was observed in L6 and L18. The explained variability was 23.1% and 32.1%, (Figure 5a and Figure 5e, respectively). In the constructed PLS-DA model, for the L6 group, the R2 and Q2 values were 0.654 and 0.238, respectively, employing the 1st principal component, and for the L18 group, the values were 0.977 and 0.434, employing the 5th principal component. Nevertheless, at L12, the experimental groups did not show clear differentiation.

However, between the L6 and L18 groups, the metabolites that had the greatest influence on the comparison of the groups were different. As shown in Figure 5b, random forest analysis showed, in due of the VIP scores, that sphingomyelin, cholesterol, and omega-3 are the main metabolites that influence the separation of animals that ate cherry and VH, while in L18 they are ornithine, glutamate, and sphingomyelin (Figure 5f).

In the liver, although the model was not significant, a clear grouping and separation of the animals that consumed LC in L6 and L18 with respect to their respective VH was observed (Figure 6a,e). In the case of animals exposed to L12, both the LC and nLC groups seem to differ from the VH group (Figure 6c). The explained variability in L6, L12, and L18 was 27%, 15.9%, and 20.1%, respectively. Considering the random forest analysis and the VIP scores, the metabolites that had the greatest weight in the separation and differentiation of the groups were sphingomyelin, isoleucine, and free cholesterol in L6, L12, and L18, respectively, showing once again the differential effect according to the exposed photoperiod (Figure 6b,d,f).

## 4. Discussion

In this study, we investigated the effects of cherry consumption from two distinct origins on rats sensitive to photoperiods. We examined the impact of cherry intake both within and outside their typical consumption season on the FA profile, gene expression of key enzymes, and transporters involved in lipid metabolism in muscle and liver, as well as on hepatic and serum metabolites.

Consumption of cherries during the L18 season, irrespective of their origin, results in a reduction in saturated fatty acids (SFAs) in the liver. This effect is primarily attributed to a decrease in palmitic acid (PA), while animals consuming cherries exhibit an increased content of c11-18:1.PA is synthesized de novo by the *Fas1* and is later modified by successive steps of elongation and/or desaturation. In this regard, *Scd1* is key to introducing the first *cis* bond at position 9 of PA to form palmitoleic acid [31]. It has been reported that palmitoleic acid remains at low concentrations in hepatocytes, as a consequence of two processes: its rapid β-oxidation or formation to c11-18:1 by elongation [31]. In the present study we did not observe differences in the proportion of palmitoleic acid, although there was a higher level of c11-18:1 in those who consumed cherries at L18. We could infer that the lower amount of PA in the treated animals is due to the fact the metabolism is driven to a greater establishment and synthesis of c11-18:1. Notably, the L18-LC group also presented a higher flow of *Scd1*, estimated by the palmitoleic acid/PA ratio, and higher gene expression, supporting this hypothesis. In agreement with our results, other authors have described that the activity of *Scd1* is associated with an increase in hepatic mRNA levels [32], which is associated with increased lipogenesis, hepatic steatosis, fat accumulation, and metabolic disorders. However, these consequences are associated with increased OA but not c11-18:1 [33]. Other studies have observed an inverse correlation between c11-18:1 content in blood cells and myocardial risk and lipogenesis suppression [34,35]. On the other hand, although total hepatic oleate strongly correlates with TAGs accumulation in the liver, as well as with higher adipose tissue and OA content in circulating TAGs, an increase in PA does not exhibit these correlations [33]. In L12, animals that consumed cherries, regardless of origin, showed a lower amount of OA in the liver than their respective VH. Some authors assert that OA induces autophagy in hepatocytes [36], while others suggest that due to the high lipotoxicity it produces in cells, it is rapidly esterified to TAGs [37]. In this regard, our results suggest that cherry consumption at L12 would lead to lower toxicity and predisposition to autophagy compared to VH consumption. However, further studies, such as the analysis of TAG composition or autophagic enzyme activity, are required to clarify this. In addition, it could also be argued that these changes could also be influenced by factors such as differences in overall energy intake, variations in feeding behavior, and metabolic adaptations to photoperiod rather than cherry polyphenols alone.

At the muscular level, the L18-LC group, tended to have a higher expression of the FA transporter FAT/CD36 and a higher 15:0 content with respect to its VH. In contrast, they presented a lower SA. Recently, some authors have positively associated muscle 15:0 levels with increased glucose uptake, improved insulin sensitization in muscle cells, and a lower incidence of diabetes [38]. However, high SA content is asiated with serious metabolic pathologies [39,40]. Nevertheless, such an effect was not observed in the group supplemented with nLC at L18, indicating the influence of the different phenolic contents in both fruits. Interestingly, animals exposed to L6 or L12 showed a slightly greater difference in SA than their respective VH, despite the absence of a photoperiod effect. These results provide evidence of the importance of fruit consumption during the harvesting season.

MUFAs and PUFAs do not have the same lipotoxic and dysfunctional effects as long-chain SFAs; instead they promote good metabolic health by mitigating the adverse effects of SFAs [41]. In muscle, for example, unsaturated FAs antagonize the excessive production of free oxygen radicals (ROS) produced by SFAs [42], promote the synthesis of neutral TAGs [43], allow greater oxidation of SFAs [44], and prevent loss of muscle mass [45]. In this sense, the animals from the L18-LC group presented a higher palmitoleic acid and c11-18:1 content in the muscle compared to their respective VH. It has been observed that the incubation of muscular cells with palmitoleic acid antagonizes the morphological, biochemical and functional mitochondrial changes produced by SFAs [41]. Therefore, these results suggest that the origin of cherry and its (poly)phenolic profile may differentially affect the metabolism and transport of FAs from the liver to muscle.

In contrast, the animals that consumed cherries at L12 presented less palmitoleic acid than their respective VH in the muscle, whereas the c11-18:1 content did not change. Notably, photoperiod affected the proportion of palmitoleic acid, as the VH groups significantly differed in their content depending on the photoperiod, which was higher at L12. Furthermore, the proportion of palmitoleic acid in animals treated with cherries at L12 and L18 was similar. Therefore, it could be inferred that cherry consumption modulates MUFAs levels, mainly palmitoleic acid, regardless of the photoperiod. Other authors have observed in hepatic cell cultures that (poly)phenols cause a decrease in MUFAs, mainly palmitoleic acid [9]. However, animals exposed to L6 did show changes between treatments, suggesting that exposure to 12 h of light and darkness had a more significant effect on palmitic acid levels than the other photoperiods. However, further research is needed to elucidate the underlying mechanisms.

L18-nLC animals showed a higher hepatic proportion of cis PUFAs and higher *n*-6 PUFAs levels than their respective VH. Specifically, they present more LA and c11, c14-20:2. However, they also have a higher proportion of ALA and 22:5 *n*-3 and a lower expression of *Fads2*, which is key for PUFA synthesis. Pathway analysis revealed alterations in LA metabolism among the experimental groups. In agreement with our results, other authors observed that hepatocytes treated with delphinidin-3-*O*-glucoside reduced the expression of *Fads2* and the level of EPA [46]. The difference in anthocyanidin content between the different types of cherries could determine this differential effect. Furthermore, considering the evaluated hepatic hydro- and lipophilic metabolites, the L18-nLC group also had higher total cholesterol, free cholesterol, total phospholipids, phosphatidylethanolamine, phosphatidylcholine, sphingomyelin, and asparagine than their respective VH. Sphingolipids, glycerolipids, and phospholipids are bioactive membrane constituents that form lipid rafts that regulate their fluidity and serve as first and/or second messengers in different pathways [47]. Condensation of serine with PA is the first step in ceramide formation. Phosphatidylcholine and sphingomyelin are involved in several metabolic pathways [21]. Considering that all animals received the same nutritional composition of FAs, the differential effect on the content of PUFAs as well as on the amount of hepatic hydro- and lipophilic metabolites would be given by the different types of cherries and the season in which they were consumed. In this sense, nLC has a higher amount of flavonols, specifically quercetin, than LC does. Flavonols modulate the expression of desaturases, lipogenic enzymes, and transcription factors in hepatocytes and adipocytes [8]. However, there is limited evidence on the crosstalk between (poly)phenol intake, circadian rhythm, and hepatic FA profiles. Considering the different metabolic pathways in which these lipid substances are involved, future studies on the composition of phospholipids, glycerolipids, TAGs, VLDL, and hepatic lipoproteins are needed to elucidate the molecular and biochemical mechanisms involved.

At the muscular level, a clear effect of seasonal treatment was observed, as both groups that ingested cherries had lower amounts of total PUFAS than their respective VH. Although there were no significant differences in the different FAs, the L18-LC animals tended to have lower muscle DHA than the VH animals. Furthermore, increased gene expression of adiponectin receptor, *AdipoR2*, was observed in these animals. In agreement with our results, it has been observed that anthocyanins, which are present in greater quantities in LC, could have preventive effects on metabolic disorders in animal models because of their influence on pathways that are mainly involved in adiponectin and AMPK activation [48]. Through its receptors, adiponectin activates AMPK and PPAR, resulting in a greater uptake of blood glucose through increased translocation of the GLUT4 transporter towards the membrane surface and increased β-oxidation of FAs [49]. This modulates insulin sensitivity and affects the lipid and glucose homeostasis. Animals with L18-nLC tend to have a lower proportion of EPA and DHA and a higher expression of Cs, which are crucial for the oxidative metabolism of muscle FAs. Considering that *n*-3 PUFAs are predominantly oxidized above other FAs [50], it can be inferred that cherry consumption, regardless of origin, increases the oxidation of *n*-3 PUFAs. Additionally, other authors have demonstrated that proanthocyanidin consumption improves AMPK phosphorylation in obese rats [51], although further studies are needed to clarify the underlying mechanisms. In this study, animals exposed to L12 that ingested cherries had higher levels of total cis PUFAs, mainly PUFAS *n*-6, than their respective VH. There was a clear effect of photoperiod, since the VH of L12 and L18 were also affected. It is worth noting that the animals treated in L12 exhibited values comparable to those of the groups supplemented in L18, further underscoring the impact of consuming fruit outside its harvest and consumption season.

## 5. Conclusions

In this study, we investigated the effects of cherry consumption from different origins and its intake under different photoperiods on the FA profile and the expression of key enzymes and transporters involved in lipid metabolism in the liver and muscle of Fischer 344 rats. Hydrophilic and lipophilic serum and hepatic metabolites were also analyzed in this study. Our findings indicate that the consumption of cherries in season, regardless of their origin, reduces hepatic SFA content, mainly PA, correlated with increased unsaturation mediated by *Scd1*. In addition, the L18-nLC group showed changes in LA metabolism compared to that of the VH group. During L12, animals that consumed cherries showed a higher proportion of liver and muscle SFAs, mainly SA, as well as reduced *Scd1* enzymatic activity. A major strength of this study is the evaluation of hepatic and muscular fatty acid profiles and metabolomic changes under different photoperiods, which provides a comprehensive view of the chrononutritional effects of cherry consumption. The use of two cherry types with distinct (poly)phenolic profiles and the inclusion of photoperiods allowed us to identify season- and composition-dependent metabolic responses. However, many observed associations showed only marginal statistical significance (0.05 < *p* < 0.1). Future research incorporating sex-specific analyses could provide a clearer understanding of these effects, potentially revealing sex-related differences in lipid metabolism regulation in response to cherry consumption. Additionally, validating these findings in human cohorts will be essential to fully elucidate the underlying mechanisms.

## Figures and Tables

**Table 1:** Liver fatty acid profile of Fischer 344 rats fed a standard diet determined by GC-FID and enzymatic flux.

	L6			L12			L18			2XA
	VH	LC	nLC	VH	LC	nLC	VH	LC	nLC	
Σ PUFAs	33.84 ± 1.75 ^ab^	36.91 ± 1.31 ^a^	35.05 ± 0.96 ^ab^	32.08 ± 0.51 ^b^	36.87 ± 1.70 ^a^	36.04 ± 0.90 ^ab^*	32.39 ± 0.89 ^b^	33.52 ± 1.66 ^ab^	36.28 ± 0.68 ^a^	T
Σ PUFAs *n*-6	27.53 ± 1.49 ^ab^	30.08 ± 1.20 ^a^	28.47 ± 0.85 ^ab^	26.76 ± 0.48 ^ab^	30.06 ± 1.53 ^a^	29.44 ± 0.88 ^a^	25.99 ± 0.79 ^b^	26.99 ± 1.28 ^a^*^b^	29.72 ± 0.61 ^a^	T
LA	10.68 ± 0.38 ^ab^*	11.21 ± 0.95 ^ab^	10.83 ± 0.28 ^ab^	12.32 ± 0.78 ^b^	11.52 ± 0.53 ^ab^	11.10 ± 0.67 ^ab^	9.61 ± 0.43 ^a^	10.27 ± 0.49 ^a^	12.30 ± 0.78 ^b^	PxT*
GLA	0.09 ± 0.01 ^a^	0.10 ± 0.01 ^ab^	0.11 ± 0.0 ^ab^	0.09 ± 0.01 ^a^	0.10 ± 0.01 ^ab^*	0.12 ± 0.0 ^b^	0.11 ± 0.01 ^ab^	0.11 ± 0.0 ^ab^	0.10 ± 0.01 ^ab^	
20:2	0.17 ± 0.01 ^a^	0.20 ± 0.01 ^a^	0.20 ± 0.01 ^a^*^b^	0.19 ± 0.01 ^a^*^b^	0.20 ± 0.02 ^b^	0.19 ± 0.02 ^ab^	0.16 ± 0.01 ^a^	0.18 ± 0.01 ^a^	0.21 ± 0.01 ^b^	T
DGLA	0.73 ± 0.06	0.71 ± 0.13	0.69 ± 0.06	0.63 ± 0.03	0.71 ± 0.05	0.68 ± 0.05	0.72 ± 0.0	0.78 ± 0.12	0.62 ± 0.09	
AA	15.39 ± 1.34 ^ab^	17.33 ± 0.47 ^ab^	16.28 ± 0.60 ^ab^	12.88 ± 0.54 ^b^	16.99 ± 1.01 ^a^	16.79 ± 0.19 ^a^	15.22 ± 0.23 ^ab^	15.16 ± 1.36 ^ab^	15.89 ± 0.82 ^ab^	T*
Σ PUFAs *n*-3	6.31 ± 0.28 ^a^	6.84 ± 0.16 ^a^	6.58 ± 0.27 ^a^	5.42 ± 0.17 ^b^	6.81 ± 0.18 ^a^	6.60 ± 0.07 ^a^	6.40 ± 0.16 ^a^	6.53 ± 0.40 ^a^	6.55 ± 0.28 ^a^	T, PxT*
ALA	0.14 ± 0.02 ^a^	0.16 ± 0.04 ^a^	0.17 ± 0.01 ^a^	0.25 ± 0.05 ^b^	0.19 ± 0.02 ^ab^	0.16 ± 0.03 ^a^	0.14 ± 0.02 ^a^	0.15 ± 0.03 ^ab^*	0.23 ± 0.03 ^b^	PxT*
EPA	0.37 ± 0.02 ^ac^	0.38 ± 0.06 ^a^	0.32 ± 0.01 ^ab^	0.28 ± 0.02 ^bc^*	0.32 ± 0.02 ^ab^	0.30 ± 0.02 ^b^	0.36 ± 0.01 ^c^	0.37 ± 0.02 ^ac^	0.28 ± 0.02 ^b^	P*, T
22:5	0.91 ± 0.06	0.99 ± 0.04	1.00 ± 0.11	0.92 ± 0.07	0.98 ± 0.05	0.95 ± 0.08	0.80 ± 0.07	0.97 ± 0.13	1.01 ± 0.07	
DHA	4.75 ± 0.23 ^a^	5.16 ± 0.10 ^a^	4.94 ± 0.23 ^a^	3.92 ± 0.10 ^b^	5.13 ± 0.19 ^a^	4.99 ± 0.06 ^a^	5.0 ± 0.20 ^a^	4.87 ± 0.34 ^a^	4.91 ± 0.23 ^a^	T, PxT
ENZYMATIC FLUX										
*SCD1* ^1^	0.20 ± 0.02 ^a^*^b##^	0.16 ± 0.02 ^a##^	0.18 ± 0.00 ^a^	0.19 ± 0.01 ^ab^	0.15 ± 0.01 ^a#^	0.15 ± 0.01 ^ab#^	0.17 ± 0.01 ^ab^	0.20 ± 0.01 ^b##^	0.17 ± 0.01 ^ab^*	
*SCD1* ^2^ OA/SA	0.75 ± 0.11 ^a^	0.60 ± 0.04 ^a^	0.68 ± 0.05 ^a^	1.03 ± 0.13 ^b^	0.66 ± 0.05 ^a^	0.68 ± 0.02 ^a^	0.65 ± 0.03 ^a^	0.77 ± 0.14 ^a^	0.81 ± 0.11 ^ab^	PxT
∆6D	0.01 ± 0.00 ^ab^	0.01 ± 0.00 ^ab^	0.01 ± 0.0 ^a^*^b^	0.01 ± 0.0 ^b^	0.01 ± 0.00 ^a^*^b^	0.01 ± 0.00 ^a^	0.01 ± 0.0 ^ab^	0.01 ± 0.0 ^ab^	0.01 ± 0.0 ^a^*^b^	PxT*
∆5D	21.37 ± 2.37	26.56 ± 4.36	24.02 ± 2.10	21.08 ± 0.26	24.43 ± 2.38 *	25.32 ± 2.24	21.62 ± 0.38	20.23 ± 2.08 *	27.10 ± 3.47 *	
DHA/ALA	36.52 ± 6.17 ^a^	37.78 ± 8.61 ^a^	30.08 ± 2.68 ^ab^	18.85 ± 3.36 ^b^	27.30 ± 2.86 ^ab^	34.96 ± 6.97 ^a^	39.19 ± 7.15 ^a^	37.05 ± 8.45 ^a^	23.58 ± 4.59 ^ab^	PxT*
EPA/ALA	2.88 ± 0.62 ^b^	3.04 ± 1.06 ^ab^	1.96 ± 0.15 ^ab^	1.34 ± 0.31 ^b^	1.71 ± 0.16 ^ab^	2.09 ± 0.44 ^ab^	2.86 ± 0.58 ^a^	2.79 ± 0.57 ^a^	1.35 ± 0.31 ^a^*^b^	
AA/LA	1.45 ± 0.13 ^ab^*	1.58 ± 0.12 ^a^	1.50 ± 0.05 ^ab^	1.12 ± 0.12 ^b^	1.47 ± 0.05 ^ab^*	1.53 ± 0.07 ^a^	1.59 ± 0.06 ^a^**	1.50 ± 0.17 ^a^	1.32 ± 0.14 ^ab^**	PxT*
Elongase	0.91 ± 0.12 ^ab^	1.18 ± 0.13 ^ab^	1.02 ± 0.03 ^ab^	1.02 ± 0.16 ^ab^**	1.30 ± 0.14 ^a^	1.18 ± 0.10 ^a^**	0.87 ± 0.07 ^ab^**	0.91 ± 0.10 ^b^	1.19 ± 0.12 ^ab^**	T*

Proportion of fatty acids (FAs) expressed as % FAMEs and enzymatic flux of FAs biosynthesis in liver of animals exposed to different photoperiods (L6, L12, and L18 correspond to 6, 12, and 18 h of light per day, respectively) and supplemented with treatment: Local Cherry (LC), non-Local Cherry (nLC), or vehicle (VH). Σ PUFAs: polyunsaturated fatty acids; LA: Linoleic acid; GLA: gamma-linolenic acid; 20:2: c11,c14-20:2; DGLA: Dihomo-γ-linolenic acid; AA: arachidonic acid; ALA: α-Linolenic acid; EPA: Eicosapentaenoic acid; 22:5: c7,c10,c13,c16,c19-22:5; DHA: Docosahexaenoic acid; *Scd1* ^1^, Stearoyl-CoA desaturase 1, Palmitoleic acid/PA; *Scd1* ^2^ OA/SA, Stearoyl-CoA desaturase 1: oleic acid/stearic acid; ∆6D, delta-6 desaturase, GLA/LA; ∆5D, delta-5 desaturase, AA/DGLA; Elongase, c11-18:1/palmitoleic acid. Values expressed as mean ± SEM (*n* = 4). P, photoperiod; T, treatment; PxT, interaction of photoperiod and treatment. (Two-way ANOVA (2xA), *p* < 0.05); different letters (a, b, c) indicate significant statistical differences *p* < 0.05; * indicates trend 0.05 < *p* < 0.1 (post hoc DMS, one-way ANOVA). ** Indicates trend (0.05 < *p* < 0.1) in the same photoperiod. # Indicates significant statistical differences *p* < 0.05 and ## indicates trend 0.05 < *p* < 0.1 (Student’s *t*-test).

**Table 2:** Muscle fatty acid profile of Fischer 344 rats fed a standard diet determined by GC-FID.

	L6			L12			L18			2XA
	VH	LC	nLC	VH	LC	nLC	VH	LC	nLC	
Σ PUFAs	40.13 ± 1.74 ^a^	38.81 ± 3.26 ^ab^	43.45 ± 0.21 ^a^	34.67 ± 1.64 ^c^	39.63 ± 0.83 ^b^	39.60 ± 0.40 ^b^	42.19 ± 0.52 ^a^	38.73 ± 0.88 ^b^	39.24 ± 1.26 ^b^	P, PxT
Σ PUFAs *n*-6	27.66 ± 0.64 ^a^	25.79 ± 2.62 ^a^	27.65 ± 1.43 ^a^	25.15 ± 0.95 ^b^	27.16 ± 0.39 ^a^	27.36 ± 1.21 ^a^	28.46 ± 0.17 ^a^	27.18 ± 0.47 ^a#^	27.20 ± 0.85 ^a^	P, PxT*
LA	16.66 ± 0.19 ^a^	15.53 ± 0.32 ^a^*^b^	15.48 ± 0.53 ^a^*^b^	16.21 ± 0.39 ^a^	16.23 ± 0.24 ^a^	14.54 ± 0.51 ^b^	16.86 ± 0.47 ^a^	16.48 ± 0.66 ^a^	16.14 ± 0.49 ^a^	P*, T
GLA	0.03 ± 0.01 ^b^	0.04 ± 0.01 ^ab^	0.05 ± 0.01 ^a^	0.04 ± 0.0 ^ab^*	0.04 ± 0.0 ^ab^	0.03 ± 0.0 ^a^*^b^	0.04 ± 0.01 ^ab^	0.04 ± 0.01 ^ab^	0.04 ± 0.01 ^ab^	
DGLA	0.33 ± 0.03	0.37 ± 0.02	0.39 ± 0.00	0.30 ± 0.04	0.35 ± 0.01	0.33 ± 0.03	0.35 ± 0.02	0.34 ± 0.03	0.33 ± 0.02	
20:2	0.11 ± 0.01 ^b^	0.13 ± 0.01 ^ab^	0.14 ± 0.02 ^a^	0.12 ± 0.01 ^ab^	0.12 ± 0.01 ^ab^	0.12 ± 0.0 ^ab^	0.13 ± 0.0 ^ab^	0.13 ± 0.0 ^a#^	0.13 ± 0.01 ^a^	T*
AA	9.53 ± 0.67 ^a^	11.36 ± 0.02 ^a^	10.54 ± 1.37 ^ab^*	7.46 ± 0.80 ^b^	9.35 ± 0.38 ^ab^*	10.50 ± 0.15 ^a^	10.03 ± 0.32 ^a^	9.22 ± 0.83 ^ab^	9.63 ± 0.57 ^ab^	
22:4	0.53 ± 0.02	0.60 ± 0.03	0.57 ± 0.06	0.47 ± 0.05	0.52 ± 0.02	0.54 ± 0.06	0.51 ± 0.01	0.53 ± 0.03	0.55 ± 0.0	
22:5(*n*-6)	0.48 ± 0.04 *	0.60 ± 0.04	0.62 ± 0.08 *	0.47 ± 0.07	0.54 ± 0.02	0.48 ± 0.08	0.54 ± 0.03	0.47 ± 0.03	0.51 ± 0.04	
Σ PUFAs *n*-3	12.46 ± 1.10 ^a^	13.02 ± 0.82 ^a^	14.38 ± 0.32 ^a^	11.14 ± 1.70 ^b^	12.47 ± 0.45 ^a^	10.82 ± 1.53 ^b^	13.73 ± 0.46 ^a^	11.55 ± 0.49 ^ab#^	12.04 ± 0.52 ^ab^	P, PxT
ALA	0.29 ± 0.04	0.21 ± 0.03	0.21 ± 0.01	0.33 ± 0.05	0.26 ± 0.01	0.27 ± 0.05	0.27 ± 0.04	0.32 ± 0.06	0.31 ± 0.05	
EPA	0.12 ± 0.01 ^ab^	0.13 ± 0.01 ^ab^	0.14 ± 0.00 ^a^	0.11 ± 0.02 ^ab^	0.13 ± 0.01 ^ab^	0.10 ± 0.01 ^b^	0.13 ± 0.01 ^ab^	0.12 ± 0.01 ^ab^	0.11 ± 0.0 ^a^*^b ##^	
22:5(*n*-3)	2.35 ± 0.20	2.81 ± 0.14	2.70 ± 0.41	2.21 ± 0.37	2.42 ± 0.12	2.26 ± 0.33	2.49 ± 0.08	2.39 ± 0.25	2.32 ± 0.14	
DHA	9.78 ± 0.84 ^ab^	9.87 ± 0.76 ^ab^	10.95 ± 0.11 ^a^	8.50 ± 1.38 ^b^	9.65 ± 0.36 ^ab^	8.19 ± 1.22 ^b^	10.85 ± 0.44 ^a^	8.80 ± 08 ^a^*^b^	9.30 ± 0.44 ^ab^	

Proportion of PUFAs in muscle expressed as % FAMEs of animals exposed to different photoperiods (short, L6; standard, L12; long, L18, with 6, 12 and 18 h of light, respectively) and supplemented with treatment: Local Cherry (LC), non-Local Cherry (nLC), or vehicle (VH); Σ PUFAs: polyunsaturated fatty acids; LA: Linoleic acid; GLA: gamma-linolenic acid; DGLA: Dihomo-γ-linolenic acid; 20:2: c11,c14-20:2; AA: arachidonic acid; 22:4: c7,c10,c13,c16-22:4; 22:5(*n*-6): c4,c7,c10,c13,c16-22:5; ALA: α-Linolenic acid; EPA: Eicosapentaenoic acid; 22:5(*n*-3): c7,c10,c13,c16,c19-22:5; DHA: Docosahexaenoic acid. Values expressed as mean ± SEM (*n* = 4). P, photoperiod; T, treatment; PxT, photoperiod and treatment interaction. (Two-way ANOVA (2xA), *p* < 0.05); different letters indicate significant statistical differences *p* < 0.05; * indicates trend 0.05 < *p* < 0.1 (post hoc DMS, one-way ANOVA). # Indicates significant statistical differences *p* < 0.05 and ## indicates trend 0.05 < *p* < 0.1 (Student’s *t*-test) with respective VH.

## Data Availability

Dataset available on request from the authors.

## Supplementary Materials

The following supporting information can be downloaded at: https://www.mdpi.com/article/10.3390/nu18020345/s1. Table S1. Nucleotide sequences of primers used for real time quantitative PCR. Table S2. Liver fatty acid profile of Fischer 344 rats fed a standard diet. Table S3. Muscle fatty acid profile of Fischer 344 rats fed a standard diet. Table S4. Pathway analysis of liver FA profile of nLC and LC vs VH in L18 determined by to Metaboanalyst software. Table S5. Metabolites (hydro and lipophilic) identified by nuclear magnetic resonance. Figure S1. Pathway analysis based on data from liver twenty-seven FA of L18-LC vs L18-VH groups. References [28,29,30,52] are cited in Supplementary Materials.

## Abbreviations

SFAs	Saturated Fatty Acids
MUFAs	Monounsaturated Fatty Acids
PUFAs	Polyunsaturated Fatty Acids
20:2	c11,c14-20:2 (Eicosadienoic acid)
22:4	c7,c10,c13,c16-22:4 (Docosatetraenoic acid)
22:5(*n*-6)	c4,c7,c10,c13,c16-22:5 (Docosapentaenoic acid, *n*-6)
22:5(*n*-3)	c7,c10,c13,c16,c19-22:5 (Docosapentaenoic acid, *n*-3)
AA	Arachidonic Acid
ALA	α-Linolenic Acid
DGLA	Dihomo-γ-linolenic Acid
DHA	Docosahexaenoic Acid
SA	Stearic Acid
EPA	Eicosapentaenoic Acid
GLA	Gamma-Linolenic Acid
LA	Linoleic Acid
OA	Oleic Acid
PA	Palmitic Acid
*Scd1* 1	Stearoyl-CoA Desaturase 1
*Scd1* 2 OA/SA	Stearoyl-CoA Desaturase 1
∆6D	Delta-6 Desaturase
∆5D	Delta-5 Desaturase
FAs	Fatty Acids
FAMEs	Fatty Acid Methyl Esters
GC-FID	Gas Chromatography–Flame Ionization Detection
VH	Vehicle (control)
LC	Local Cherry
nLC	non-Local Cherry
L6	Short photoperiod (6 h of light)
L12	Standard photoperiod (12 h of light)
L18	Long photoperiod (18 h of light)
P	Photoperiod
T	Treatment
PxT	Interaction of Photoperiod and Treatment
