# Supplementary material for: Chrononutritional Effects of Cherry Consumption on Hepatic Lipid Profile
Source: Nutrients. 2026 Jan 21;18(2):345. doi: 10.3390/nu18020345 (PMC12844997; doi:10.3390/nu18020345)
Supplement: Supplementary file 1 [file nutrients-18-00345-s001.zip › nutrients-4016064-supplementary.pdf]

Supplementary Material

Hepatic and muscular gene expression analysis.

Liver and gastrocnemius muscle total RNA was extracted using TriPure reagent (Roche Diagnostic, Sant Cugat del Vallès, Barcelona, Spain) according to the manufacturer's protocol. cDNA was synthesized by reverse transcription using High-Capacity cDNA Reverse Transcription (Thermo Fischer Scientific, Illkirch-Graffenstaden, France). Specific cDNA amplification was performed by real-time polymerase chain reaction (RT-qPCR) using iTaq Universal SYBR Green Supermix (Bio-Rad, Barcelona, Spain). Eight samples per group were used (n=8). Different primers related to hepatic metabolism of FA and muscle lipid metabolism, obtained from Biomers.net (Ulm, Germany), (Supplementary Table 1): Acc2, Acetyl-CoA carboxylase 2; AdipoR2, Adiponectin receptor 2; CS, Citrate Synthase; Elovl2, Elongation of very-long-chain fatty acid enzyme 2; Fads1, Δ-5 desaturase; Fads2, Δ-6 desaturase; FAT/cd36, fatty acid translocase, homologue of CD36; Scd1, Δ-9 desaturase. The relative expression of each mRNA level was calculated as a percentage of the L18 group using the Pfaffl method [1].

Supplementary Table S1. Nucleotide sequences of primers used for real time quantitative PCR.

Gene	Forward Primer (5′ to 3′)	Reverse Primer (5′ to 3′)	Tissue
Acc2	CTTTTCTAGGTCCCCGAGTGA	CTTCCGCTCCAGGGTAGAGTT	M
AdipoR2	CCACACAACACAAGAATCCG	CCCTTCTTCTTGGGAGAATGG	M
Cs	CCGTGCTCATGGACTTGGGCCTT	CCCCTGGCCCAACGTAGATGCTC	M
Elovl2	TTTGGCTGTCTCATCTTCCA	GGGAAACCATTCTTCACTTC	L
Fads1	TACAGGCAACCTGCAACGTTC	GGTGCCACCTTGTGGTAGTTGT	L
Fads2	GGAACCATCGACATTTCCAG	TCTTTATGTCGGGGTCCTTG	L
FAT/cd36	GTCCTGGCTGTGTTTGGA	GCTCAAAGATGGCTCCATTG	M
Scd1	GAGGAACATCATTCTCATGGTCC	CGTACACGTCATTCTGGAACG	L

The table shows the nucleotide sequences of primers used for PCR amplification in liver (L) and gastrocnemius muscle (M) tissues. Acc2, Acetyl-CoA carboxylase 2; AdipoR2, Adiponectin receptor 2; CS, Citrate Synthase; Elovl2, Elongation of very-long-chain fatty acid enzyme 2; Fads1, Δ-5 desaturase; Fads2, Δ-6 desaturase; FAT/CD36, fatty acid translocase, homologue of CD36; Scd1, Δ-9 desaturase.

Hepatic and muscle lipid extraction and gas chromatogram analysis.
Briefly, 400 mg or 500 mg of either muscle or liver, respectively, were weighted. The samples were homogenized together with chloroform, methanol and water maintaining the ratio 1:2:0.8. After addition of 1 mL of chloroform and 1 mL of water (ratio 2:2:1.8), and vortex and centrifugation, the organic phase was separated and dried under stream of N2. For the determination of lipid profile, derivation of FA was done by methylation of methanol potassium hydroxide. FA methyl esters (FAMEs) determination was carried out by gas chromatography (Shimadzu (GC-2014) equipped with FID detector and using the CP-Sil 88 (100 m × 0.25 mm × 0.2 μm, film thickness, Varian, Lake Forest, CA, USA). Four samples per group were used (n=4). FAMEs were identified by comparing their retention relative times of commercial standards, and the chromatogram analysis was done with the LabSolution Shimadzu Software. Values were expressed as a percentage of the total FAMEs [2] .
Enzymatic flow of key enzymes in the biosynthesis of unsaturated FA was estimated by determining the relationship between products and substrates from FAMEs, following the ratios described by Sain, J [3].

Extraction of serum and liver metabolites for NMR analysis and data processing.
Briefly, 50 mg of liver were mixed with 1 mL of water:acetonitrile (H2O:CH3CN: 1:1 v/v). The resulting solution was centrifuged (19000 g, 15 min, T= 4 °C) and the aqueous supernatant was carefully transferred to a fresh eppendorf. To obtain the lipophilic phase, 1 mL of chloroform:methanol solution (CHCl 3 /CH 3OH 2:1 T=0 ºC) was added. Then, it was vortexed, homogenized and after 10 minutes of incubation at room temperature, it was centrifuged. The aqueous residues were removed under N2 stream and the hepatic lipid supernatant was obtained. Eight samples per group were used (n=8).
On the other hand, for the extraction of the polar and lipophilic metabolites of the serum, 200 mL of sample were mixed with 400 μL of methanol and 100 μL of water and after vortexing, 200 μL of chloroform was added. Once obtained a monophasic solution, 600 μL chloroform and 300 μL water were added, centrifuged (19000 x g, 4 ºC, 15 min) to obtain the aqueous supernatant (upper phase) and the lipid supernatant (bottom phase). Both extracts were subjected to a N2 stream to dryness and stored at -20 ºC up to further analysis.
The dried hydrophilic and lipophilic extracts were reconstituted in 600 μL of deuterium oxide (D2O) phosphate buffer (0.05 mM PBS, pH 7.4, 99.5% D2O)

with 0.73 mM trisilylpropionic acid (TSP) and in a deuterated chloroform (CDCl 3) / deuterated methanol (CD3OD) (2:1) solution with 1.18 mM tetramethylsilane (TMS), respectively for NMR measurement. Extracts were transferred to 5 mm glass NMR tubes for analysis. The detailed technical characteristics of the equipment can be consulted in the procedure carried out by Palacio et al. [4].

The acquired 1H NMR spectra were compared to references of pure compounds from the metabolic profiling AMIX spectra database (Bruker), HMDB, Chenomx NMR suite 8.4 software (Chenomx Inc., Edmonton, AN, Canada) and databases for metabolite identification. In addition, we assigned metabolites by 1H–1H homonuclear correlation (COSY and TOCSY) and 1H–13C heteronuclear (HSQC) 2D NMR experiments and by correlation with pure compounds run in-house. After pre-processing, specific 1H NMR regions identified in the spectra were integrated using the AMIX 3.9 software package.

Supplementary table S2. Liver fatty acid profile of Fischer 344 rats fed a standard diet.

Fatty Acid	L6			L12			L18			2xA
	VH	LC	nLC	VH	LC	nLC	VH	LC	nLC	
Σ SFA	43.46±0.28a*	43.10±0.55a	43.32±0.55a	41,87±0.96a*	42.59±0.74a	43.38±0.36a*	46.27±0.34b	43.48±0.72a	41.89±0.30a	P, T*, PxT
14:0	0.86±0.10ab	0.74±0.04a*b	0.87±0,02ab	1.00±0,02a	0.74±0.07b	0.76±0.04b	0.93±0.03ab	0.92±0.09a	0.88±0.07ab	T
15:0	0.15±0.02	0.15±0.01	0.17±0.01	0.17±0.01	0.17±0.01	0.16±0.01	0.16±0.01	0.18±0.01	0.19±0.01	
PA	29.40±0.85ab*	27.89±0.72a	29.08±0.58ab	29.00±0.38a	27.58±0.89a	28.48±0.27a	31.42±0.53b	29.52±0.92ab*	28.43±0.27a	P*, T
17:0 ISO	0.28±0.01	0.27±0.04	0.29±0.02	0.26±0.01	0.27±0.03	0.26±0.01	0.37±0.02	0.31±0.02	0.27±0.01	
17:0	0.20±0.04	0.24±0.01	0.23±0.01	0.23±0.02	0.25±0.02	0.25±0.01	0.22±0.01	0.22±0.03	0.25±0.01	
SA	12.85±0.64ab*c	14.07±0.53a	12.95±0.18ab	11.57±0.61b	13.84±0.20a	13.69±0.47a	13.52±0.23c	12.60±0.81a*bc	12.11±0.55bc*	PxT
Σ MUFAs	19.65±2.02ab**	18.47±1.16a**	19.91±0.42ab	22.82±1.41b	19.10±0.98a	19.18±0.69a	19.51±0.46a	21.33±1.42ab	20.45±0.77a	PxT*
c9-14:1	0.07±0.02	0.05±0.01	0.07±0.00	0.08±0.02	0.05±0.01	0.05±0.01	0.07±0.00	0.06±0.02	0.05±0.01	
Palmitoleic acid	5.92±0.70b	4.44±0.64a	5.15±0.07ab	5.34±0.69ab	4.17±0.40a	4.29±0.33a	5.46±0.35ab	5.97±0.45b	4.70±0.36ab*	T*
c9-17:1	0.19±0.03	0.21±0.01	0.22±0.01	0.24±0.03	0.24±0.01	0.22±0.01	0.24±0.01	0.23±0.00	0.23±0.01	
c6-18:1	0.09±0.01	0.11±0.02	0.09±0.01	0.09±0.01	0.09±0.01	0.10±0.01	0.15±0.02	0.14±0.02	0.10±0.01	
OA	9.46±0.89a	8.40±0.36a	8.81±0.53a	11.71±1.03b	9.04±0.60a	9.29±0.29a	8.75±0.27a	9.39±1.05a	9.59±0.90a	
c11-18:1	4.89±0.05ab	5.00±0.22ab	5.28±0.23ab	5.11±0.10ab	5.25±0.11ab	4.98±0.19b	4.67±0.16b	5.29±0.21a	5.47±0.17a	T*,PxT*
c12-18:1	0.17±0.03	0.19±0.01	0.20±0.02	0.17±0.01	0.17±0.01	0.17±0.02	0.16±0.02	0.19±0.02	0.20±0.02	
c11-20:1	0.09±0.01ab	0.08±0.00a	0.09±0.0a*b	0.10±0.01b	0.10±0.01b	0.09±0.01ab	0.08±0.01a	0.08±0.00a	0.10±0.01b	P*, PxT
Σ PUFAs-cis	33.84±1.75ab	36.91±1.31a	35.05±0.96ab	32.08±0.51b	36.87±1.70a	36.04±0.90ab*	32.39±0.89b	33.52±1.66ab	36.28±0.68a	T
Σ PUFAs n-6	27.53±1.49ab	30.08±1.20a	28.47±0.85ab	26.76±0.48ab	30.06±1.53a	29.44±0.88a	25.99±0.79b	26.99±1.28a*b	29.72±0.61a	T
LA	10.68±0.38ab*	11.21±0.95ab	10.83±0.28ab	12.32±0.78b	11.52±0.53ab	11.10±0.67ab	9.61±0.43a	10.27±0.49a	12.30±0.78b	PxT*

GLA	0.09±0.01a	0.10±0.01ab	0.11±0.00ab	0.09±0.01a	0.10±0.01ab*	0.12±0.00b	0.11±0.01ab	0.11±0.00ab	0.10±0.01ab	
20:2 n-6	0.17±0.01a	0.20±0.01a	0.20±0.01a*b	0.19±0.01a*b	0.20±0.02b	0.19±0.02ab	0.16±0.01a	0.18±0.01a	0.21±0.01b	T
DGLA	0.73±0.06	0.71±0.13	0.69±0.06	0.63±0.03	0.71±0.05	0.68±0.05	0.72±0.00	0.78±0.12	0.62±0.09	
AA	15.39±1.34ab	17.33±0.47ab	16.28±0.60ab	12.88±0.54b	16.99±1.01a	16.79±0.19a	15.22±0.23ab	15.16±1.36ab	15.89±0.82ab	T*
22:2 n-6	0.10±0.03	0.10±0.03	0.10±0.03	0.09±0.01	0.09±0.02	0.11±0.03	0.08±0.06	0.14±0.03	0.15±0.02	
22:4 n-6	0.37±0.04	0.42±0.03	0.44±0.04	0.41±0.00	0.45±0.04	0.46±0.05	0.41±0.01	0.39±0.02	0.45±0.03	
Σ PUFAs n-3	6.31±0.28a	6.84±0.16a	6.58±0.27a	5.42±0.17b	6.81±0.18a	6.60±0.07a	6.40±0.16a	6.53±0.40a	6.55±0.28a	T, PxT*
ALA	0.14±0.02a	0.16±0.04a	0.17±0.01a	0.25±0.05b	0.19±0.02ab	0.16±0.03a	0.14±0.02a	0.15±0.03ab*	0.23±0.03b	PxT*
EPA	0.37±0.02ac	0.38±0.06a	0.32±0.01ab	0.28±0.02bc*	0.32±0.02ab	0.30±0.02b	0.36±0.01c	0.37±0.02ac	0.28±0.02b	P*, T
22:3 n-3	0.09±0.00	0.10±0.01	0.12±0.01	0.08±0.00	0.12±0.01	0.11±0.01	0.12±0.01	0.11±0.01	0.09±0.01	
22:5 n-3	0.91±0.06	0.99±0.04	1.00±0.11	0.92±0.07	0.98±0.05	0.95±0.08	0.80±0.07	0.97±0.13	1.01±0.07	
DHA	4.75±0.23a	5.16±0.10a	4.94±0.23a	3.92±0.10b	5.13±0.19a	4.99±0.06a	5.00±0.20a	4.87±0.34a	4.91±0.23a	T, PxT
Σ NI	1.07±0.15	0.99±0.04	0.90±0.11	0.73±0.08	0.95±0.06	0.93±0.10	0.99±0.04	0.89±0.05	0.78±0.09	

Proportion of fatty acids in liver expressed as % FAME of animals exposed to different photoperiods (short, L6; standard, L12; long, L18, with 6, 12 and 18 hours of light, respectively) and supplemented with treatment: Local cherry (LC), non-Local cherry (nLC) or vehicle (VH). Σ NI: sum of unidentified fatty acids; ΣSFA: saturated fatty acids ΣMUFAs: monounsaturated fatty acids; ΣPUFAs: polyunsaturated fatty acids; AA: arachidonic acid: c4,c8,c11,c14-20:4 n-6; ALA: α-Linolenic acid: c9,c12,c15-18:3 n-3; DGLA: Dihomo-γ-linolenic acid: c8,c11,c14-20:3 n-6; DHA: Docosahexaenoic acid: c4,c7,c10,c13,c16,c19-22:6 n-3; SA: Stearic acid: 18:0; EPA: Eicosapentaenoic acid: c5,c8,c11,c14,c17- 20:5 n-3; GLA: gamma-linolenic acid: c6, c9,c12-18:3 n-6; LA: Linoleic acid: c9,c12-18:2 n-6; OA: Oleic Acid: c9-18:1; PA; Palmitic acid: 16:0; 20:2 n-6: c11,c14-20:2; 22:2 n-6: c13,c16-22:2; 22:4 n-6: c7,c10,c13,c16-22:4; 22:5 n-3: c7,c10,c13, c16,c19- 22:5. Values expressed as mean ± SEM (n=4). P, photoperiod; T, treatment; PxT, photoperiod and treatment effect. (two-way ANOVA (2xA), $p < 0.05$); different letters indicate significant statistical differences $p < 0.05$; * indicates trend $0.05 < p < 1.0$

(Post-hoc DMS, one way ANOVA). ** Indicates trend within pars. # Indicates significant statistical differences $p < 0.05$ and ## indicates trend $0.05 < p < 0.1$ (Student´s t-test).

Supplementary table S3. Muscle fatty acid profile of Fischer 344 rats fed a standard diet

Fatty Acid	L6			L12			L18			2xA
	VH	LC	nLC	VH	LC	nLC	VH	LC	nLC	
Σ SFA	39.39±0.44b	41.30±0.56a	40.32±0.37ab*	40.05±0.16b	40.51±0.29ab**	41.31±0.92a**	39.22±0.24ab	40.83±0.07ab	39.89±0.45b	T, PxT*
4:0	0.32±0.07	0.47±0.11	0.48±0.05	0.28±0.06	0.33±0.13	0.53±0.07	0.26±0.03	0.35±0.11	0.33±0.03	
12:0	0.11±0.06	0.08±0.00	0.07±0.01	0.08±0.02	0.07±0.01	0.11±0.02	0.07±0.00	0.08±0.01	0.07±0.01	
14:0	0.83±0.06a	0.78±0.04a	0.70±0.02a	1.09±0.19b	0.90±0.08ab	0.80±0.02a	0.81±0.00a	0.96±0.09ab	0.91±0.06ab	
15:0	0.13±0.01	0.16±0.02	0.14±0.02	0.14±0.01	0.15±0.01	0.14±0.00	0.14±0.01*	0.17±0.01*	0.16±0.01	
PA	28.08±0.74a	29.64±0.60a*b	29.38±0.59ab	29.91±0.61c	29.22±0.42ac**	30.63±0.53ac**	28.47±0.46abc*	29.36±0.53ab	28.57±0.45b	P
17:0	0.18±0.02ab*	0.20±0.01ab	0.21±0.02ab	0.18±0.01a	0.19±0.01ab	0.19±0.02ab	0.22±0.00b	0.21±0.01ab	0.22±0.01ab	P
SA	9.30±0.50a	9.35±0.41a	8.91±0.55ab	8.04±0.84b	9.15±0.21a	8.42±0.91a	9.83±0.17a	8.70±0.64b	9.17±0.39ab*	PxT
20:0	0.02±0.01	0.03±0.01	0.03±0.00	0.03±0.01	0.04±0.01	0.04±0.02	0.03±0.01	0.03±0.01	0.05±0.03	
22:0	0.03±0.01a	0.03±0.01a	0.05±0.02ab	0.01±0.00a**	0.02±0.00a	0.05±0.02ab**	0.02±0.00a	0.03±0.02ab*	0.06±0.03b	T
24:0	0.03±0.00	0.04±0.01	0.02±0.01	0.01±0.00	0.02±0.00	0.05±0.01	0.01±0.00	0.02±0.01	0.04±0.02	
Σ MUFAs	18.15±1.18ab	16.23±0.54a	14.62±0.43a	20.01±4.56b	18.68±0.67ab	18.29±1.66b	16.65±0.77a	19.40±0.94ab	19.67±1.53ab	P

c9-14:1	0.04±0.01	0.04±0.01	0.04±0.00	0.08±0.03	0.06±0.01	0.40±0.33	0.05±0.00	0.07±0.01	0.06±0.01	
c7-16:1	0.26±0.02	0.27±0.01	0.26±0.01	0.27±0.01	0.27±0.02	0.31±0.03	0.27±0.00	0.30±0.01	0.28±0.01	
Palmitoleic Acid	3.15±0.26a	2.79±0.15a	3.05±0.50a	4.76±1.02b	3.68±0.36ab#	2.96±0.27a#	3.08±0.18a	3.91±0.37ab##	3.62±0.40ab	
c9-17:1	0.12±0.02	0.11±0.01	0.11±0.01	0.16±0.02	0.15±0.02	0.12±0.04	0.15±0.01	0.19±0.01	0.18±0.01	
c6-18:1	0.08±0.01	0.10±0.02	0.10±0.01	0.09±0.01	0.13±0.02	0.13±0.03	0.11±0.02	0.12±0.01	0.11±0.03	
OA	10.10±1.04ab*	8.53±0.39ab	7.22±0.28b	13.39±1.46c	9.79±0.38ab	10.98±1.93ac	8.49±0.53ab	9.96±0.60a	10.68±1.25ab	
c11-18:1	4.25±0.08ab	4.23±0.09a	4.20±0.06a	4.44±0.11ab	4.42±0.05ab	4.33±0.15ab	4.34±0.07ab	4.65±0.06c	4.56±0.18bc	P
c12-18:1	0.07±0.01	0.08±0.02	0.07±0.01	0.07±0.00	0.08±0.00	0.03±0.02	0.09±0.01	0.09±0.00	0.09±0.01	
c11-20:1	0.08±0.01	0.08±0.00	0.08±0.00	0.10±0.01	0.09±0.01	0.08±0.01	0.08±0.01	0.09±0.01	0.10±0.02	
Σ PUFAs	40.13±1.74a	38.81±3.26ab	43.45±0.21a	34.67±1.64c	39.63±0.83b	39.60±0.40b	42.19±0.52a	38.73±0.88b	39.24±1.26b	P,PxT
ΣPUFAs n-6	27.66±0.64a	25.79±2.62a	27.65±1.43a	25.15±0.95b	27.16±0.39a	27.36±1.21a	28.46±0.17a	27.18±0.47a	27.20±0.85a	P, PxT*
LA	16.66±0.19a	15.53±0.32a*b	15.48±0.53a*b	16.21±0.39a	16.23±0.24a	14.54±0.51b	16.86±0.47a	16.48±0.66a	16.14±0.49a	P*. T
GLA	0.03 ± 0.01b	0.04 ± 0.01ab	0.05 ± 0.01a	0.04±0.00ab*	0.04±0.0ab	0.03± 0.0a*b	0.04 ± 0.01 ab	0.04 ± 0.01ab	0.04±0.01ab	
DGLA	0.33±0.03	0.37±0.02	0.39±0.00	0.30±0.04	0.35±0.01	0.33±0.03	0.35±0.02	0.34±0.03	0.33±0.02	
20:2 n-6	0.11±0.01b	0.13±0.01ab	0.14±0.02a	0.12±0.01ab	0.12±0.01ab	0.12±0.0ab	0.13±0.00ab	0.13±0.00a	0.13±0.01a	T*
AA	9.53±0.67a	11.36±0.02a	10.54±1.37ab*	7.46±0.80b	9.35±0.38ab*	10.50±0.15a	10.03±0.32a	9.22±0.83ab	9.63±0.57ab	

22:4 n-6	0.53±0.02	0.60±0.03	0.57±0.06	0.47±0.05	0.52±0.02	0.54±0.06	0.51±0.01	0.53±0.03	0.55±0.00	
22:5 n-6	0.48±0.04*	0.60±0.04	0.62±0.08*	0.47±0.07	0.54±0.02	0.48±0.08	0.54±0.03	0.47±0.03	0.51±0.04	
ΣPUFAs n-3	12.46±1.10a	13.02±0.82a	14.38±0.32a	11.14±1.70b	12.47±0.45a	10.82±1.53b	13.73±0.46a	11.55±0.49ab	12.04±0.52ab	P, PxT
ALA	0.29±0.04	0.21±0.03	0.21±0.01	0.33±0.05	0.26±0.01	0.27±0.05	0.27±0.04	0.32±0.06	0.31±0.05	
EPA	0.12±0.01ab	0.13±0.01ab	0.14±0.0a	0.11±0.02ab	0.13±0.01ab	0.10±0.01b	0.13±0.01ab	0.12±0.01ab	0.11±0.00a*b##	
22:5 n-3	2.35±0.20	2.81±0.14	2.70±0.41	2.21±0.37	2.42±0.12	2.26±0.33	2.49±0.08	2.39±0.25	2.32±0.14	
DHA	9.78±0.84ab	9.87±0.76ab	10.95±0.11a	8.50±1.38b	9.65±0.36ab	8.19±1.22b	10.85±0.44a	8.80±0.28ab	9.30±0.44ab	
ΣNI	1.13±0.21	1.52±0.25	1.34±0.12	1.01±0.17	1.35±0.11	1.67±0.35	1.08±0.10	1.25±0.24	1.12±0.03	

Proportion of fatty acids in gastrocnemius muscle expressed as % FAME of animals exposed to different photoperiods (short, L6; standard, L12; long, L18, with 6, 12 and 18 hours of light, respectively) and supplemented with treatment: Local cherry (LC), non-Local cherry (nLC) or vehicle (VH). Σ NI: sum of unidentified fatty acids; ΣSFA: saturated fatty acids ΣMUFAs: monounsaturated fatty acids; ΣPUFAs: polyunsaturated fatty acids; AA: arachidonic acid: c4,c8,c11,c14-20:4 n-6; ALA: α-Linolenic acid: c9,c12,c15-18:3 n-3; DGLA: Dihomo-γ-linolenic acid: c8,c11,c14-20:3 n-6; DHA: Docosahexaenoic acid: c4,c7,c10,c13,c16,c19-22:6 n-3; SA: Stearic acid: 18:0; EPA: Eicosapentaenoic acid: c5,c8,c11,c14,c17- 20:5 n-3; GLA: gamma-linolenic acid: c6, cis-9,c12-18:3 n-6; LA: Linoleic acid: c9,c12-18:2 n-6; OA: Oleic Acid: c9-18:1; PA; Palmitic acid: 16:0; 20:2 n-6: c11,c14-20:2; 22:2 n-6: c13,c16-22:2; 22:4 n-6: c7,c10,c13,c16-22:4; 22:5 n-3: c7,c10,c13, c16,c19- 22:5. Values expressed as mean ± SEM (n=4). P, photoperiod; T, treatment; PxT, photoperiod and treatment effect. (two-way ANOVA, $p < 0.05$); different letters indicate significant statistical differences $p < 0.05$; * indicates trend 0.05< $p < 1.0$ (Post-hoc DMS, one way ANOVA). ** Indicates trend within pars. # indicate significant statistical differences $p < 0.05$ and ## indicates trend 0.05< $p < 0.1$ (Student´s t-test) with respective VH.

Supplementary Table S4. Pathway analysis of liver FA profile of nLC and LC vs VH in L18 determined by to Metaboanalyst software.

Pathway Name	Match Status	p	-Log(p)	FDR	Impact
nLC vs VH in L18					
FA elongation	1/39	0.002	2.654	0.007*	0.0
FA degradation	1/39	0.002	2.654	0.007*	0.0
LA metabolism	1/5	0.020	1.686	0.041*	1.0
FA biosynthesis	2/47	0.025	1.594	0.041*	0.014
Biosynthesis of unsaturated FA	10/36	0.029	1.528	0.041*	0.0
ALA metabolism	1/13	0.102	0.987	0.120	0.333
AA metabolism	1/36	0.506	0.295	0.506	0.332
LC vs VH in L18					
FA elongation	1/39	0.133	0.874	0.467	0.0
FA degradation	1/39	0.133	0.874	0.467	0.0
FA biosynthesis	2/47	0.304	0.516	0.481	0.014
ALA metabolism	1/13	0.330	0.481	0.481	0.333
LA metabolism	1/5	0.343	0.463	0.481	1.0
Biosynthesis of unsaturated FA	10/36	0.516	0.287	0.602	0.0
AA metabolism	1/36	0.865	0.062	0.865	0.332

Liver twenty-seven fatty acids (FA) from animals exposed 18 hours of light (L18) and treated with nLC, LC or VH were exposed to pathway analysis using Metaboanalyst. Main metabolic pathways significantly affected, the total amount of metabolites involved in the pathway, the amount of metabolites that have information, the p value, false discovery rate (FDR), and impact, are shown. ALA: alpha-Linolenic acid; LA: Linoleic acid; AA: Arachidonic acid. p value < 0.05 and FDR < 0.05 were considered significant (*). Kyoto encyclopedia of Genes and Genomes (KEGG) was database utilized.

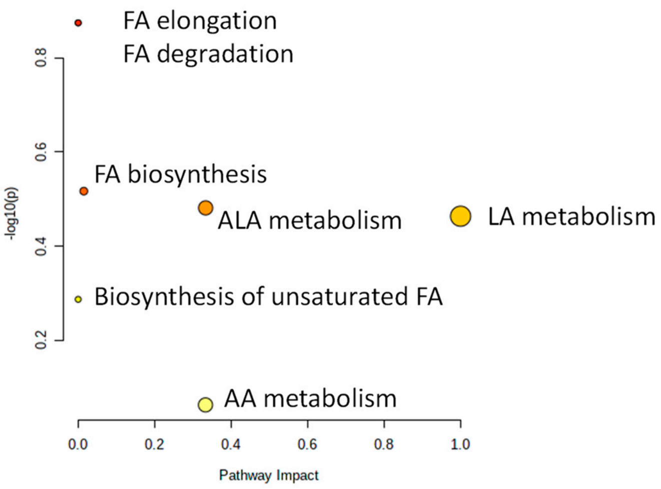

Supplementary Figure S1 Pathway analysis based on data from liver twenty-seven FA of L18-LC vs L18-VH groups. Overview of pathway analysis from Kyoto encyclopedia of Genes and Genomes (KEGG) database. AA, arachidonic acid: c4,c8,c11,c14-20:4 n-6; AL, Linoleic acid: c9,c12-18:2; ALA: α-linolenic acid: c9,c12,c15-18:3 n-3.

Supplementary Table S5. Metabolites (hydro and lipophilic) identified by nuclear magnetic resonance.

Aqueous Metabolites	Tissue	Lipidic Metabolites	Tissue
3-hydroxybutyrate	S, L	AA+EPA	S
Acetate	S, L	Diacylglycerides	S, L
Alanine	S, L	Docosahexaenoic acid	S
Allantoin	S, L	Esterified Cholesterol	S, L
Ascorbate	L	Free Cholesterol	S, L
Asparagine	S, L	Linoleic Acid	S
Aspartate	L	Monounsaturated fatty acids	S
ATP/ADP/AMP	L	Oleic Acid	S
Betaine	S, L	Omegas-3	S
Carnitine	S, L	Polyunsaturated fatty acids	S
Choline	S, L	Sphingomyelin	S, L
Citrate	S	Triacylglycerides	S, L
Creatine	S, L	Total Cholesterol	S, L
Dimethylamine	S, L	Total Fatty Acids	S, L

Dimethylglycine	S	Total Phospholipids	S, L
Formate	S, L		
Fumarate	L		
Glucose	S		
Glucose-6-Phosphate	L		
Glutamate	S, L		
Glutamine	S, L		
Glutathione	L		
Glycerol	S		
Glycine	S, L		
Glycogen	L		
Histamine	L		
Histidine	S, L		
Inosine	L		
Isoleucine	S, L		
Lactate	S, L		
Leucine	S, L		
Lysine	S, L		
Mannose	L		
Methionine	S, L		
NAD+	L		
Niacinamide	L		
Ornithine	S, L		
Phenylalanine	S, L		
Pyruvate	S		
Sarcosine	L		
Serine	S		
Succinate	S, L		
Taurine	S		
Theronine	S		
Trimethylamine	L		
Tryptophan	S, L		
Tyrosine	S, L		
UDPs	L		
UMP	L		
Uracil	L		
Uridine	L		
Valine	S, L		
Xanthine	L		

All the metabolites from serum (S) and liver (L) were obtained by performing a nuclear magnetic resonance (NMR) analysis. ATP: adenosine triphosphate; AA: Arachidonic acid; ADP: adenosine diphosphate; AMP: adenosine monophosphate; EPA: Eicosapentaenoic acid; NAD+: Nicotinamide adenine dinucleotide; UDP: Uridine diphosphate, UMP: Uridine monophosphate.

Bibliography

1. Pfaffl, M.W. Quantification strategies in real-time PCR; 2004;

2. Masson, L.; Alfaro, T.; Camilo, C.; Carvalho, a; Illesca, P.; Torres, R.; Bernal, C. Fatty acid composition of soybean / sunflower mix oil , fish oil and butterfat applying the AOCS Ce 1j-07 method. 2015, 66, 1–17, doi:10.3989/gya.0692141.

3. Saín, J. Ácidos grasos trans dietarios: interacción nutricional con las diferentes familias de ácidos grasos insaturados [Doctoral thesis, Universidad Nacional del Litoral] 2014 http://hdl.handle.net/11185/748.

4. Palacios-Jordan, H.; Martín-González, M.Z.; Suárez, M.; Aragonès, G.; Muguerza, B.; Rodríguez, M.A.; Bladé, C. The Disruption of Liver Metabolic Circadian Rhythms by a Cafeteria Diet Is Sex-Dependent in Fischer 344 Rats., doi:10.3390/nu12041085.
